# Performance of gimbal-based dynamic tumor tracking for treating liver carcinoma

**DOI:** 10.1186/s13014-018-1180-1

**Published:** 2018-12-05

**Authors:** Marc Ziegler, Tobias Brandt, Sebastian Lettmaier, Rainer Fietkau, Christoph Bert

**Affiliations:** Department of Radiation Oncology, Universitätsklinikum Erlangen, Friedrich-Alexander-Universität Erlangen-Nürnberg, Universitätsstraße 27, 91054 Erlangen, Germany

**Keywords:** Tumor tracking - stereotactic body radiation therapy - liver cancer - real-time monitoring

## Abstract

**Background:**

Since the introduction of tumor tracking in radiotherapy, it is possible to ensure a precise irradiation of moving targets. To follow the tumor movement, most systems rely on the detection of implanted markers and correlation models between the internal and external patient movement. This study reports the clinical workflow and first results of the dynamic tumor tracking (DTT) performance for patients with liver carcinoma at the Vero SBRT system of the University Hospital Erlangen regarding the detection of the internal marker and the changes of the determined correlation models.

**Methods:**

So far 13 liver patients were treated with DTT. For each patient, two fiducial markers (FM), which are monitored with X-rays during treatment, were implanted in the vicinity of the tumor. All patients received a fraction dose of 4–6 Gy with 8 to 12 fractions. Treatment and patient data is evaluated by processing the acquired log-files of the DTT treatment. Based on this, the marker detection and the changes of the correlation model between the internal and external movement is investigated.

**Results:**

The median treatment time was 19:42 min. During treatment a median of 173 X-ray stereoscopic images were acquired. The marker detection was successful in 64.6% of the images. The FM detection is independent of the relative angle between the marker and the imager, but shows a dependency on the average intensity surrounding the FM position within the kV images. The number of correlation models needed during treatment increases in the presence of baseline shifts. The comparison of the correlation models shows large differences in the internal-external correlation between the different models acquired for one patient.

**Conclusion:**

Thirteen liver patients were treated with DTT at the Vero SBRT system and the marker detection was analyzed. Furthermore, the importance of regularly monitoring the internal target motion could be shown, since the correlation between the internal and external motion changes considerably over the course of the treatment.

**Electronic supplementary material:**

The online version of this article (10.1186/s13014-018-1180-1) contains supplementary material, which is available to authorized users.

## Introduction

In radiation therapy, the precise irradiation of the target can be challenging, especially in the case of intra-fractionally moving tumors. Different techniques were developed to either reduce the tumor movement (e.g. breath holding techniques [1] or abdominal compression [2]) or to compensate for its motion during irradiation (e.g. gating [3] or dynamic tumor tracking (DTT) [4–6]). The advantages of techniques that reduce the tumor motion, are that they can be used independently of the treatment machine which provides large availability across many institutes. Tumor tracking is one of the most technologically advanced techniques. Until now there are only few commercial systems available for this treatment modality. The first system was the CyberKnife (Accuray Inc., Sunnyvale, CA, USA) [4] which consists of a small LINAC mounted on a robotic arm. The robotic arm is then used to follow the tumor motion with the LINAC. Besides this robotic tracking, further techniques were explored like MLC [5], couch [7], or gimbal tracking [8]. With the Vero stereotactic body radiation therapy (SBRT) system (Brainlab AG, Munich, Germany and Mitsubishi Heavy Industry, Tokyo, Japan) a commercial gimbal tracking system was introduced in 2009. In this system the LINAC is mounted on an O-ring with two gimbals, allowing for pan and tilt movement of the treatment beam which is subsequently used to follow the target motion. The internal tumor position is monitored by two orthogonal X-ray tubes attached to the O-ring at +/− 45° relative to the MV beam of the gantry [6].

All current DTT capable systems rely on correlation models and the detection of the tumor motion. In most cases, internal fiducial markers are used which are monitored with X-ray images to determine the internal target position. To reduce the amount of X-ray images that need to be acquired during treatment, both the CyberKnife and the Vero use a correlation model between the internal motion and a superficial surrogate motion. This correlation model is then used to predict the internal target position from the external motion signal which can be measured noninvasively and thus reducing the amount of necessary X-ray images.

One of the advantages of DTT compared with breath-hold or abdominal compression is that the patient can breathe freely during the treatment which increases patient comfort. Compared to gating, DTT is expected to have lower treatment times, because during gating the beam is only engaged for about one quarter of the breathing cycle whereas during tracking the treatment can be administered during the entire breathing cycle.

The use of SBRT for primary hepatocellular carcinoma (HCC) as well as liver metastasis has proven to be very successful with 2 year control rates of 50–100% [9–11]. To reduce safety margins, motion compensating methods such as abdominal compression [12], four-dimensional (4D) cone-beam computed tomography image guidance [13–15], computer-controlled deep-inspiration breath-hold in combination with a 4D ultrasound tracking system [16] or DTT with either MLC, robotic, couch or gimbal tracking [17] can be used.

So far thirteen patients with liver cancer were treated with the DTT procedure at the Vero system of the Universitätsklinikum Erlangen. This study will present the DTT workflow and first results of the DTT performance with a focus on marker detection rate and influence of motion parameters on the model-rebuild rate.

## Materials and methods

### Patient data

Thirteen patients with liver tumors were treated at the Vero system with DTT at the Universitätsklinikum Erlangen between 2016 and 2018. Patient characteristics are listed in Table 1. All patients were treated with a fraction dose of 6 Gy with 8 to 12 fractions resulting in a total of 132 fractions. Patient 204 is a recurrent patient and was treated twice with DTT in a period of 1.5 years.

**Table 1:** Patient data of the treated patients

ID	Age	Sex	Tumor	Volume PTV [ccm]	Plan Type	Dose [Gy]	Fractions
204	88	m	HCC G2	271.98	Conformal	60.0	10
204*	90	m	HCC	55.67	Conformal	48.0	8
206	52	m	liver metastasis	251.26	Conformal	72.0	12
207	75	m	HCC G3	52.03	Conformal	72.0	12
209	67	m	liver metastasis	1013.12	IMRT	48.0	8
210	54	m	liver metastasis	31.21	Conformal	40.0	10
211	73	w	liver metastasis	106.26	Conformal	72.0	12
212	73	w	liver metastasis	925.08	IMRT	72.0	12
213	54	m	liver metastasis	72.21	Conformal	72.0	12
214	51	m	liver metastasis	230.97	Conformal	72.0	12
215	70	m	liver metastasis	64.03	Conformal	72.0	12
216	77	m	liver metastasis	56.76	Conformal	72.0	12
217	85	m	HCC	204.53	IMRT	72.0	12

### Fiducial marker implantation and pre-treatment imaging

The ExacTrac Vero 3.5.4 software (Brainlab AG, Munich, Germany) used for the DTT treatment requires implanted fiducial radio-opaque markers (FM) in close proximity to the isocenter for the detection of the tumor movement. Two long markers (Visicoil, IBA, Schwarzenbruck, Germany; diameter: 0.75 mm, length: 20 mm (10 mm for patient 204)) were implanted one week prior pre-treatment imaging using computed tomography (CT)-guidance in close vicinity of the tumor and their endpoints defined in ExacTrac. For treatment planning, four CT scans were acquired using a Siemens Sensation Open CT scanner (Siemens Healthcare AG, Erlangen, Germany): a deep expiration scan, a deep inspiration scan, a free breathing scan and an 8-phase four-dimensional (4D) CT. Except for patient 210, every patient was intravenously given contrast agent before imaging to improve tumor visibility. The deep expiration CT and a co-registered magnetic resonance imaging (MRI) (T1-weighted DIXON sequence in expiration with contrast agent) scan were used for gross tumor volume (GTV) delineation within the iPlan RT Image 4.1.2 software (Brainlab AG, Munich, Germany). The co-registration was performed manually by the treating physician based on the FM position. The 4D CT was used during treatment planning to determine the motion amplitude of the tumor. The free breathing and inhalation scans are part of the protocol used for command based breath-hold, which was the default treatment method for liver patients before the introduction of DTT at the Vero system of the University Hospital Erlangen. Because breath-hold was used as the backup treatment technique for all DTT patients, they received the same CT scan protocol.

The planning target volume (PTV) was obtained by adding 5 mm isotropic safety margins to the GTV in end-exhale. This margin is based on the experience with breath-hold treatments at the University Hospital Erlangen which was adapted for DTT. No explicit margin reduction was performed in the transition from breath-hold to DTT. However, the PTV for a treatment in expiration is determined by a 5 mm expanded ITV-like union of the GTV in the expiration and free-breathing scan. Thus, the DTT PTV is smaller compared to the breath-hold PTV. The volume reduction was not investigated in this study since it was previously described by Depuydt et al. [17] and Matsuo et al. [18].

Treatment planning was performed in iPlan RT Dose 4.5.4 (Brainlab AG, Munich, Germany) on the acquired deep expiration CT. All patients were treated with six to eleven 6 MV photon beams. The fraction dose of 6 Gy was prescribed to the isocenter with the D_95_ surrounding the PTV. A maximum dose of 20 Gy for 800 cm^3^ of the liver was used as a clinical goal. Except for three patients, a 3D conformal radiation therapy (3DCRT) treatment plan was used since currently no end-to-end quality assurance protocol exists for DTT intensity-modulated radiation therapy (IMRT) plans which verifies the IMRT delivery in combination with gimbal movement. Nevertheless, two patients received an IMRT treatment due to very large PTV volumes and one patient due to two separate targets that were irradiated at the same time. In the case of the simultaneous irradiation of two targets, the Vero system treats them as a single target and therefore assumes that both targets are moving equally. Therefore, it was verified from the acquired CT images that both targets move in the same way over the course of the breathing cycle prior to treatment planning. Attempts using 3DCRT plans resulted in an insufficient plan quality in these cases. The IMRT plan delivery was checked by delivering it into an ArcCheck detector (SunNuclear, Melbourne, FL, USA) under stationary conditions. Before applying DTT, the DTT workflow was checked in a quality assurance procedure based on gafchromic films.

### DTT workflow

**Fig. 1:**
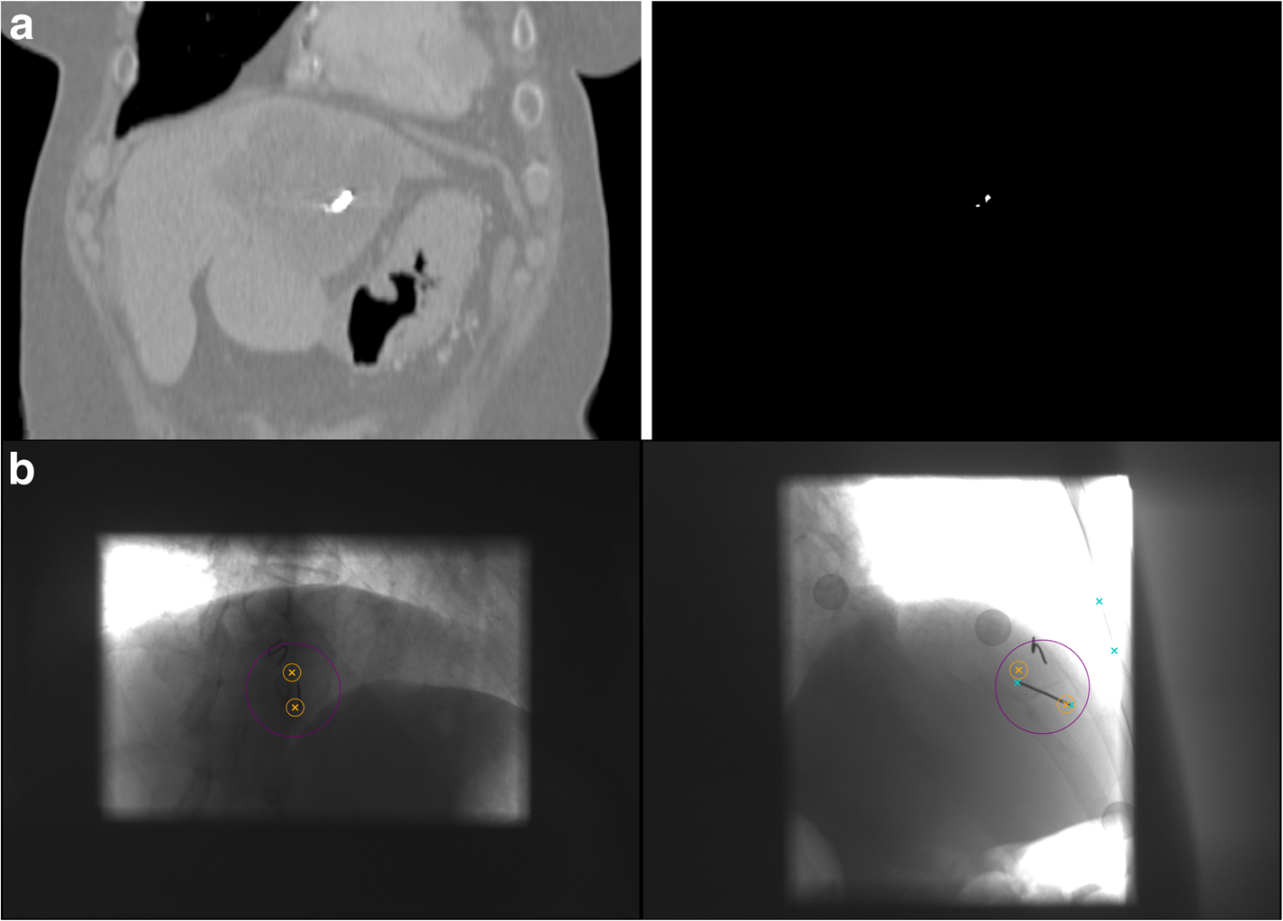
Part **a** shows an example of the extreme windowing used for the marker definition. The endpoints can be seen much clearer in the strong windowing. Part **b** shows X-ray images (window 4500, level 4500) of the ExacTrac system with the predicted marker position in orange and the possible detected positions in blue. The orange circle surrounding the predicted position has a radius of 3 mm. In this image the FM detection was only successful for *imager2* (right) with a surrounding intensity (purple circle) of over 5000. The dark borders on the edges come from the partly closed jaws of the imagers. The second marker visible within the image was not defined within the ExacTrac since it is strongly bent. Thus there is no predicted position for this marker

Prior treatment, the implanted markers are manually defined on the planning CT in the ExacTrac system. The definition of the endpoints of the marker can be challenging due to strong metal artifacts. By choosing an extreme windowing, such that only the marker itself remains visible (i.e. window 400, level 2300, see Fig. 1a), the impact of the artifacts can be reduced. Starting from patient 214 (including 204*) all FM definitions were performed with the aid of strong windowing.

After positioning of the patient on the treatment couch, several individual infrared (IR) markers are placed on the patient’s rib-cage (body markers) by attaching them to adhesive pads remaining on the patient for the entire treatment. In addition, an IR marker pad, consisting of a silicone-like pad with four IR markers, is placed on the abdomen following marks defined on the first treatment fraction. After the first fraction the body markers can be used for prepositioning the patient while the marker pad is used to determine the patients’ superficial breathing motion. Following prepositioning, two orthogonal X-ray images are acquired for a bony anatomy guided positioning of the patient. Then, a four-dimensional correlation model (4D model) is built between the movement of the FM and the external IR marker pad. To construct this model, a stereo-IR camera monitors the movement of the marker pad with 60 Hz and in parallel kV-images are taken at 3 Hz. The voltage, current and pulse duration of the two X-ray tubes are chosen individually for every patient. Common settings are E = 120 kV, *I* = 160 mA and *t* = 9 ms. Additionally to the tube settings, it is possible to change the field of view by closing jaws in front of the tubes. The jaw position is adapted for every patient, so that as much healthy tissue as possible is spared, while the FM must remain entirely within the imaged region during its trajectory.

From the kV-images the position of the fiducial markers is automatically extracted and used in combination with the position of the marker pad as an input for the 4D model. This model is used to predict the momentary position of the internal markers during treatment based on the speed and position of the IR marker pad [19]. After the successful creation of the 4D model the patient is repositioned by moving the center of gravity of the tumor trajectory to the machine isocenter. Thus the pan and tilt movement of the gimbal is equal into every direction.

During treatment, the 4D model is verified by acquiring stereo X-ray images every second and comparing the estimated to the detected marker position. If this deviation exceeds a threshold of 3 mm thrice, the treatment is automatically interrupted until the deviation falls again below this limit. If the deviation regularly exceeds the 3 mm threshold (e.g. due to changes within the breathing pattern) the model will be either updated or rebuilt. An update of the model uses the images taken during treatment and can thus be considered as “cheap” since no additional dose is applied. Since an update uses the detected FM positions from during the treatment, it can only be performed if these detections were mostly successful (70% within the last minute). The exact amount how many successful detections are necessary is decided by the fact if the Vero system is able to create a reasonable 4D model from the acquired target positions. If the model update fails, the model has to be rebuilt.

A second possibility for an automatic treatment beam interruption is if the automatic FM detection fails more than three times in a row. A successful detection of the FM requires the detection of all markers defined in the ExacTrac in both images. If the FM detection fails regularly it is possible to adjust the imager settings to improve the X-ray quality or to modify the FM definition. If it is not possible to find settings that lead to a reliable marker detection, the last possibility is to disable the *automatic beam off* function. This allows treating the patient even without the detection of the FM. If the *automatic beam off* is disabled, it is the operator’s responsibility to ensure that the quality of the 4D model is still adequate and the predicted FM position still coincides with the actual position within the kV images, since the FM are typically visible to the human eye.

### Data analysis

The data analysis was performed using several log-files of the ExacTrac system. In these log-files the motion trajectory of the IR markers, the parameters of the 4D model and the predicted and detected position of the FM are saved. From this, the breathing amplitudes and rates can be determined. As a measure for the baseline shift, the standard deviation of the end-exhale positions of the IR-markers was taken. The IR markers were used since they are used as an input for the 4D model and a baseline variation of the IR markers will impact the outcome. Furthermore, the IR marker position is available with high temporal resolution whereas the internal target position is acquired once per second if the FM detection was successful. Therefore, it is not always possible to accurately determine the baseline variation from the detected internal target position.

The treatment duration can be extracted by taking the time between the first and last entry in the corresponding session log-file representing the loading and closing of the treatment plan, respectively. The setup-time including the laser guided pre-positioning as well as the image guided positioning was defined as the time between the loading of the plan and the first creation of the motion model. The time required to build the correlation model can be extracted from the Vero system log files, but this is not possible for the model update process. Therefore the time needed for a model update had to be estimated based on the experience during treatment and is estimated as 20 s.

In combination with every kV image an additional file is saved in which either the detected marker position or in the case of a failed marker detection an error code is written (the error codes can be found in Additional file 1). From this information it is possible to extract the proportion of detected markers and the main reasons for the failing of the detection algorithm. Additionally, the predicted position as well as the corresponding deviation is stored within these files thus allowing to obtain the error of the 4D model.

Based on the predicted FM position, the angle of the every individual FM towards both of the imagers was calculated. For that, the marker main axis was defined as the straight connection between the defined endpoints and the relative angle of the marker main axis to the imager view axis *θ* was then determined by a scalar product (*θ* = 0°: imager positioned along marker main axis). Additionally, the average intensity surrounding the center of gravity of every marker and imager individually in a 1.5 cm radius within the images was calculated. A low image intensity represents a high density within the imaged material. The FM detection rate was then plotted against both the angle of the FM towards the imager *θ* as well as the surrounding image intensity *I*.

To investigate the changes of the correlation between the internal and external movement, all correlation models determined during the treatment were recalculated and compared. The model equation can be written as

$$ {F}_i={a}_i\bullet {x}^2+{b}_i\bullet x+{c}_i+{d}_i\bullet {\dot{x}}^2+{e}_i\bullet \dot{x}, $$

where *F*_*i*_ is the FM position in the *i*-direction, *x*/$\dot{x}$the position/velocity of the IR markers in AP-direction and *a*_*i*_, *b*_*i*_, *c*_*i*_, *d*_*i*_, *e*_*i*_ are the model parameters [19]. The position of the FM and the IR markers are saved relative to the isocenter and therefor are dependent on the positioning of the patient. Since the patients are repositioned after every model creation (so that the center of gravity of the tumor trajectory is in the isocenter), the marker position varies slightly between all model creations. Thus, before the recalculation, all marker positions (FM as well as IR marker) were shifted to a common baseline by subtracting their respective minimum positions to discard of the differences due to the positioning. Afterwards, for every patient individually, all models were recalculated based on the shifted datasets and applied to an artificial IR marker dataset. Since the predicted position depends on the location and velocity of the IR markers, it is necessary to apply all correlation models onto the same dataset in order to compare them. This dataset is given by

$$ x(t)={A}_{IR}\bullet {\sin}^2\left(t/T\right), $$

where *A*_*IR*_ is the average motion amplitude of the IR markers of the corresponding patient and *T* is the average breathing period of the patient. After applying all recalculated correlation models onto *x*(*t*), the predicted movement amplitudes of the internal marker were compared to determine the differences in the correlation between the internal and external movement. This comparison was performed for every movement direction individually, as well as for the 3D motion amplitude.

All statistical analyses in this study were performed using either linear regression or the Pearson correlation coefficient *r*. A *p*-value of less than 0.05 was considered as significant.

## Results

### Duration of DTT treatment

The median total duration of a DTT treatment was 19:42 min. Of this time, a median of 07:01 min was needed for patient positioning, including laser guided and image guided positioning. During the treatment the correlation model has to be rebuilt with an average probability of 38% and the building of the correlation model took on median 02:11 min. An update of the correlation model was performed with an average probability of 58%. Based on this information, and an estimated model update time of 20 s, the median period of validity for every 4D model can be determined by (T_treatment_ - T_setup_ - n_build_* T_build_ - n_update_* T_update_) / (n_build_+ n_update_) =4:50.

### Automatic marker detection

**Fig. 2:**
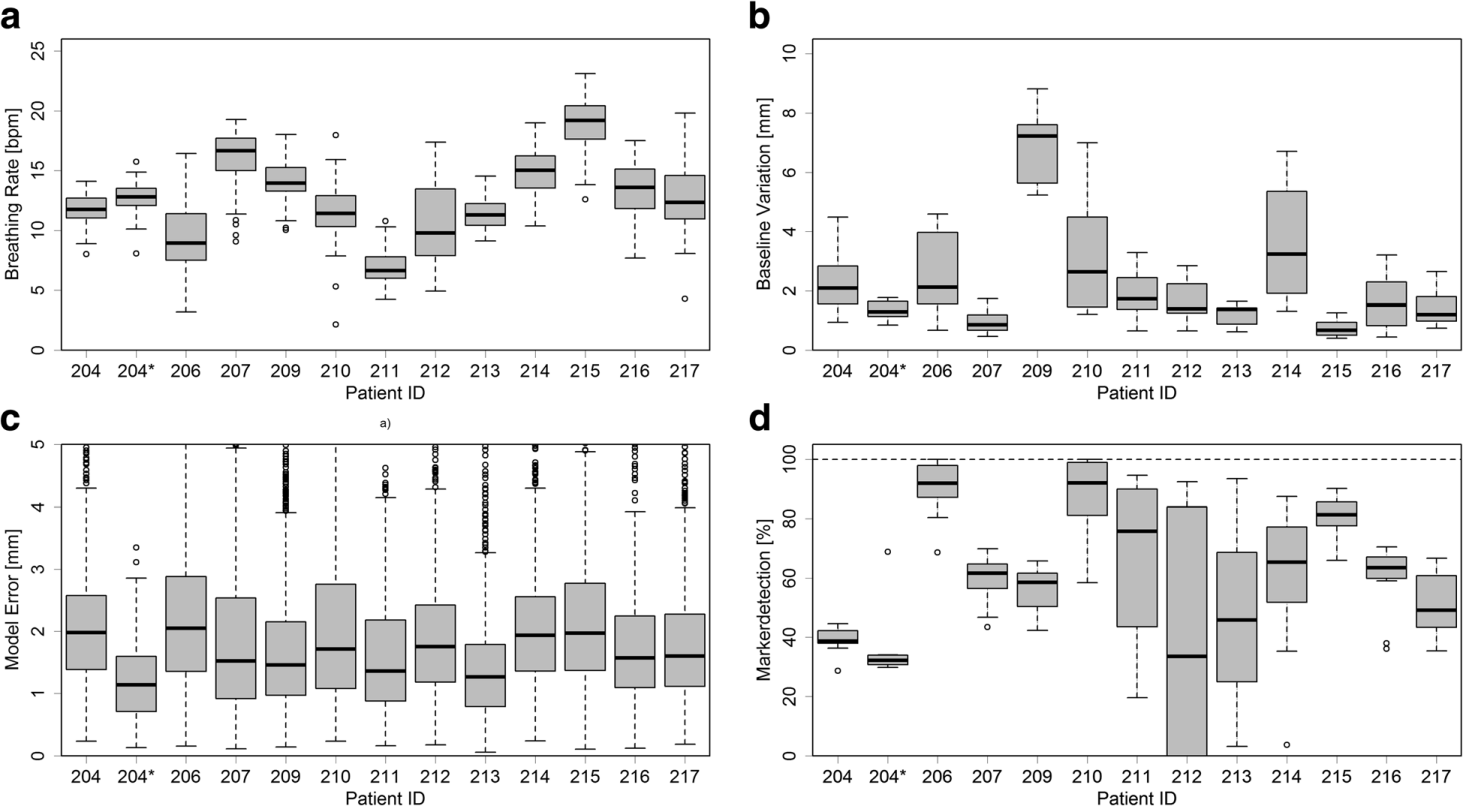
Parts **a**, **b** and **c** show the breathing characteristics of the patients via boxplots of the 3D FM motion amplitude, the breathing rate in bpm and the baseline variation in mm. **d** shows the patient specific marker detection rate over all treatment fractions. It can be seen that for some patients the detection rate shows a large day-to-day variation

On median 173 kV stereoscopic image pairs were acquired during every treatment fraction and on median the FM were detected in 64.6% of the images over all patients. Figure 2d shows a boxplot of the patient specific marker detection rate. A correlation between the patient circumference at the isocenter, which was extracted from the planning CT, and the marker detection rate could be found (*p* = 0.007). No further correlations were found between any other patient parameters.

The marker detection was analyzed with respect to the relative angle of the gold marker towards the imager as well as in dependence on the average intensity surrounding the FM within the kV image. An example of a taken kV image and the following FM detection can be seen in Fig. 1b. Within *imager2* (right) the defined marker is detected and in good agreement with the predicted position. However, within *imager1* (left) no endpoints could be detected. Thus the FM detection failed for this image set. A second marker can also be seen in Fig. 1b which was not defined within the ExacTrac since it is strongly bent and hence lead to problems in the FM detection. The surrounding intensity within *imager1* is 1735 and 5257 in *imager2*.

**Fig. 3:**
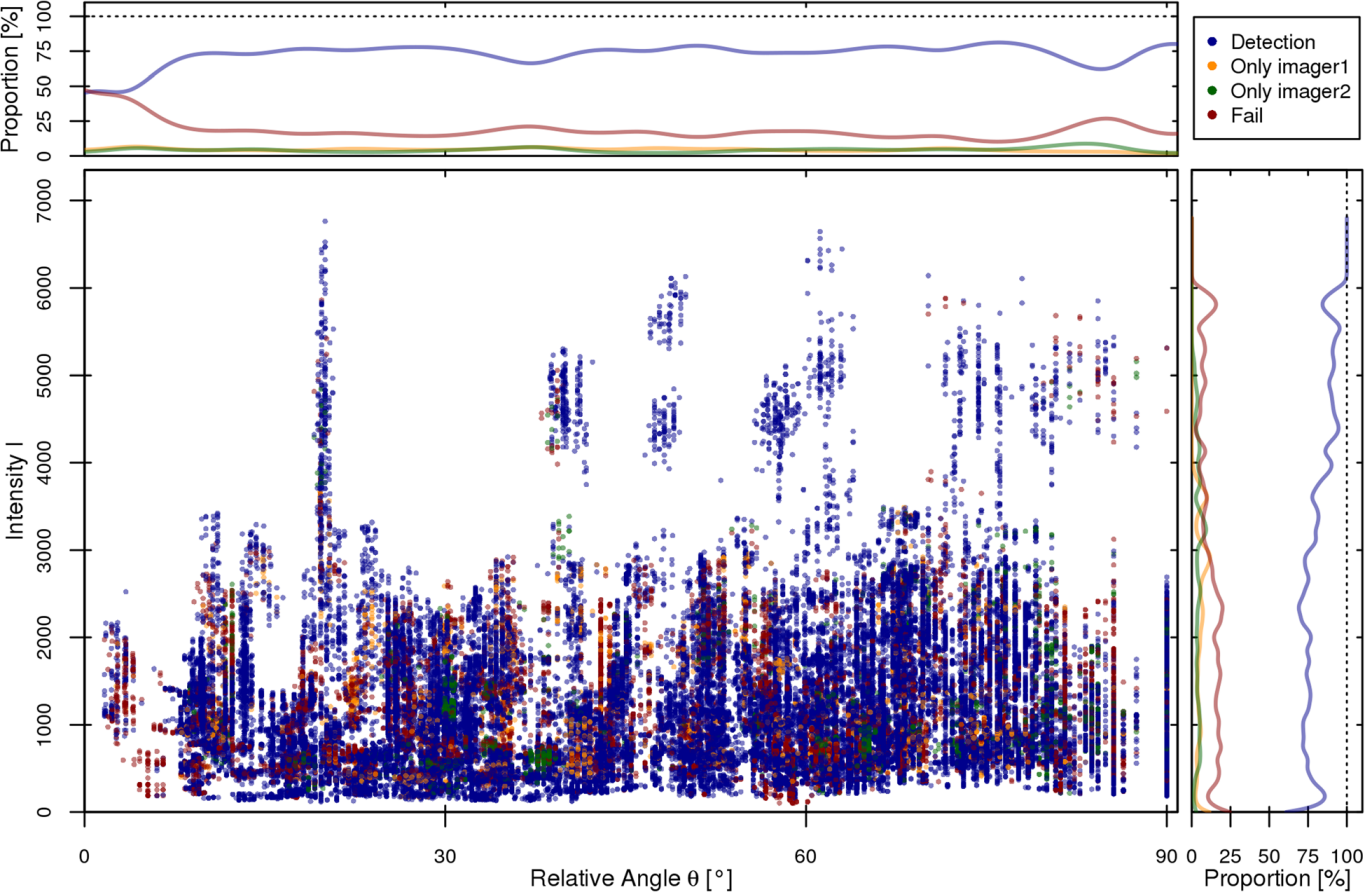
Distribution of failed (red) and successful (blue for both imagers, orange for *imager1*, green for *imager2*) FM detection depending on the angle *θ* of the marker relative to the imager as well as the average surrounding intensity *I*. Every point represent a single marker in a single imager. All points are semi-transparent for a better visualization of overlapping points. On the top and right, the relative amount of successful and failed detections is displayed

Figure 3 shows the success and failure of the FM detection depending on the relative angle *θ* and the surrounding image intensity *I*. From the resulting distribution, the relative amount of successful and failed FM detections is calculated and plotted for each dependency individually. The data show that the probability of a detection in only one of the two imagers is independent of *θ* and *I* and also no difference between *imager1* and *imager2* could be observed. The general success rate shows a clear increase for intensity values above ~ 2500 (*r* = 0.9 with *p* < 0.001 for *I* > 2500) which is in agreement with the correlation between the patient circumference and the marker detection rate. For the angular dependency, a decrease for *θ* < 10° can be observed.

To improve the marker detection the imager settings were on median changed twice during treatment by modifying the kV and mAs settings. Additionally, for patients 204, 206, 211, 212 and 213 marker definitions were changed, deleted or re-added during the treatment to obtain better marker detection. Since the strong windowing during the FM definition was introduced to the clinical workflow no marker re-definitions were necessary. Neither marker migration nor changes in the marker shape were observed.

### Motion model error

The average distance between the predicted marker position and the detected marker position during the DTT treatment is 1.67 mm with a 90th percentile of 3.33 mm. Based on a linear regression, no significant correlation between the absolute error of the 4D model with the absolute tumor motion could be observed (*p* = 0.093). Furthermore, no significant correlation between the error and the breathing rate (*p* = 0.450), the baseline variation (*p* = 0.452) or the tumor size (*p* = 0.448) could be observed. Figure 2a-c) show the patient specific breathing rate, baseline variation and 4D model error respectively.

**Fig. 4:**
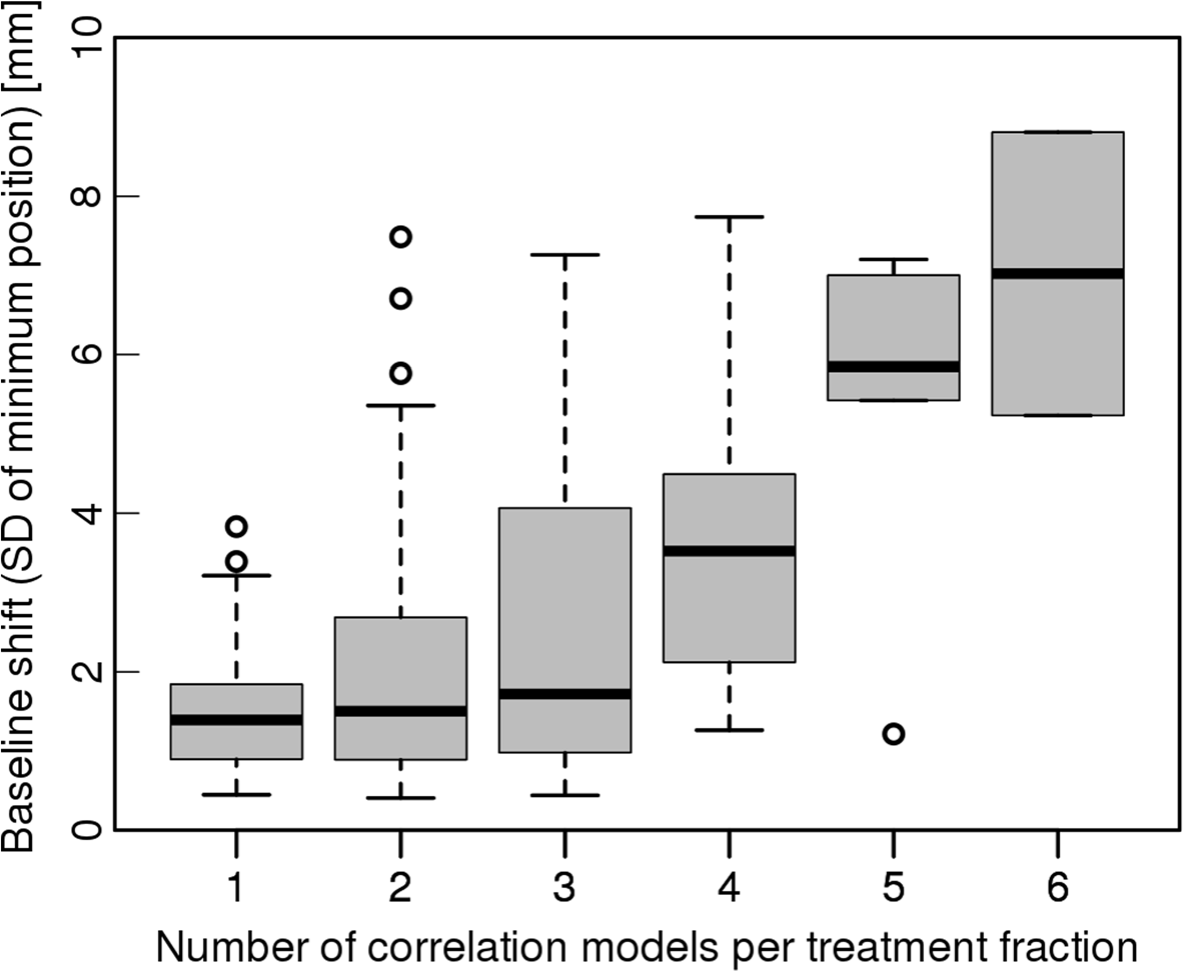
Boxplot of the baseline shifts occurring for a given number of calculated correlation models during a treatment fraction. The baseline shift is defined by the standard deviation of the end-exhale positions of the IR markers. A significant increase of the occurring baseline shifts with the number of models could be observed (*p* < 0.001)

The impact of the baseline shift on the number of correlation models used during DTT can be seen in Fig. 4. The correlation of the number of models to the baseline shift was investigated using linear regression and a significant dependency was found (*p* < 0.001). No correlation of the baseline shift to any patient parameter and no trend over the course of the treatment was found.

### Motion model analysis

**Fig. 5:**
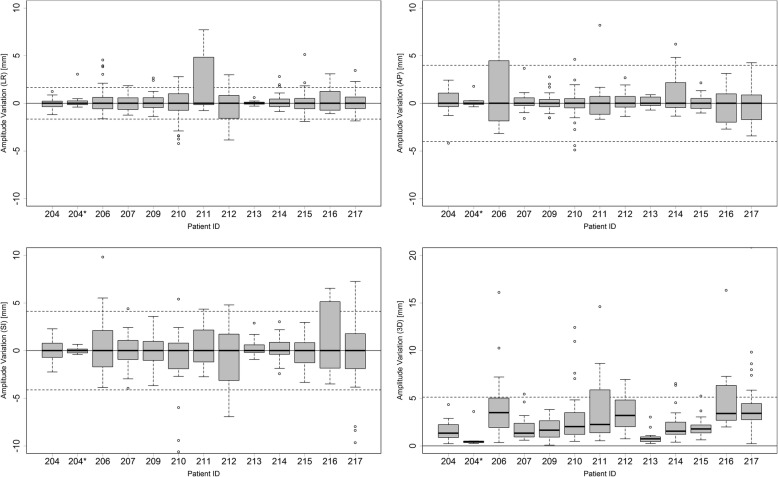
The differences of the predicted FM amplitudes relative to the median predicted amplitude (top left: LR-distances, top right: AP-distances, bottom left: SI-distances, bottom right: 3D distance). The standard deviation of 1.62 mm, 4.19 mm and 4.15 mm in the LR-, SI- and AP-direction respectively. The average 3D distance is 3.6 mm

A boxplot of the deviations between the predicted amplitudes by the different correlation models for all patients can be seen in Fig. 5. The deviations are shown for the three spatial directions and the 3D motion amplitude. All deviations are taken relative to the respective median of the patient. The dashed lines represent the standard deviations.

The comparison of the recalculated motion models revealed that the predicted tumor motion amplitudes vary with a standard deviation of 1.65 mm, 4.12 mm and 3.99 mm in the LR-, SI- and AP-direction respectively. The standard deviation of the median 3D amplitude of the FM motion is 5.12 mm. The maximum 3D distance between a predicted FM motion endpoint relative to the median predicted motion endpoint, occurring for patient 206, is 27 mm.

**Fig. 6:**
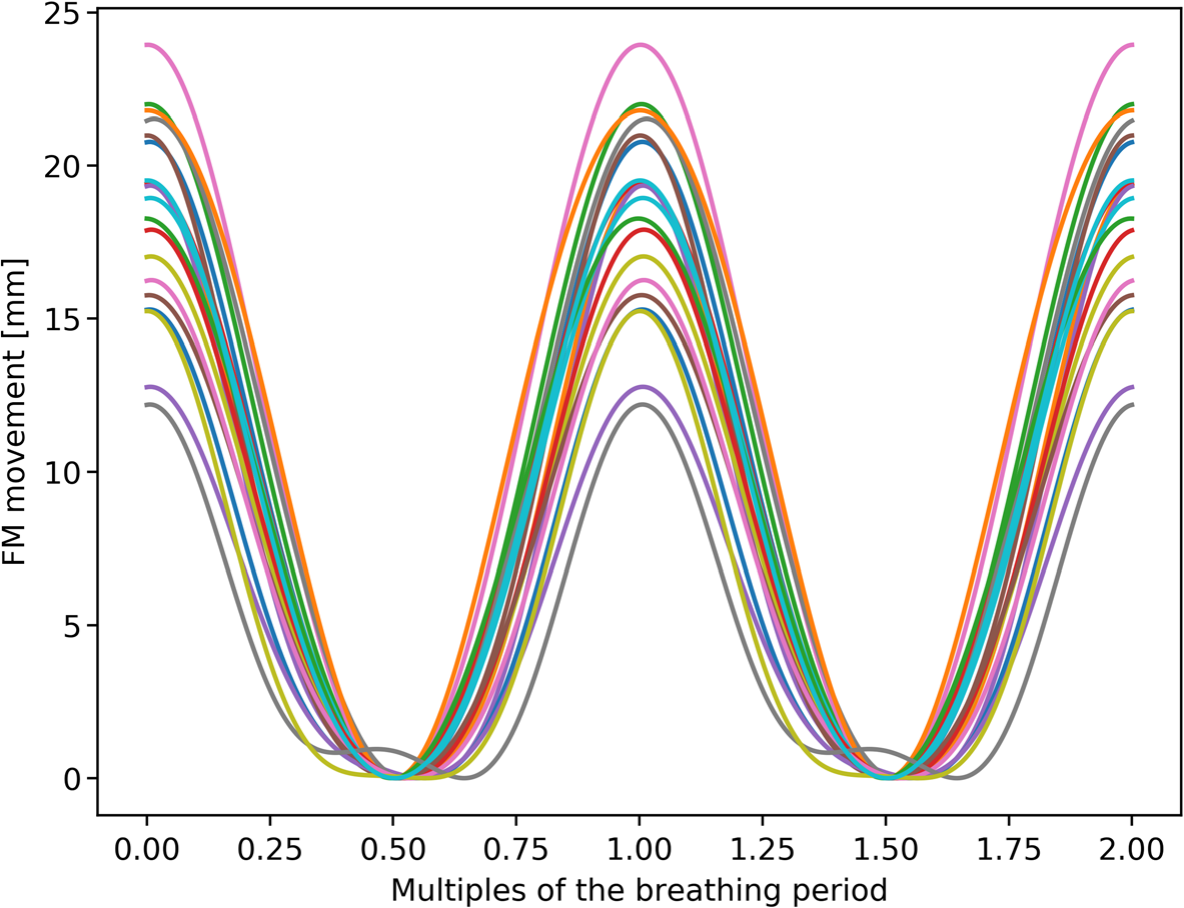
All recalculated 4D models for patient 212 applied to the artificial IR marker dataset. Every line represents the predicted FM position by one correlation model for the SI-direction

Figure 6 shows the result of applying all motion models onto the artificial IR dataset for the SI-direction for patient 212. It can be seen that the predicted tumor motion varies between 12 mm and 24 mm.

## Discussion

Until now, 13 patients could be treated with DTT at the Vero system in the University Hospital Erlangen with in total 132 fractions. Only once the treatment could not be delivered due to insufficient marker detection for the building of the 4D model. In this case, the fraction was rescheduled and delivered on the next day.

The time needed for every fraction was on median 19:42 min and thus slightly below the DTT treatment times reported in [17, 18]. Although mainly lung patients were presented by those groups, the general workflow shows no significant differences.

On average 1.38 correlation models are determined from scratch and 0.58 model updates are performed. Thus, 1.96 4D models are used on average during every treatment fraction. This value is in good agreement with a study by Matsuo et al. [18] which found that on average 1.9 4D models are calculated per treatment fraction.

The 4D model showed residual tracking errors during treatment of 1.67 mm. This error is smaller than the errors which are expected from a non-DTT treatment. For example is has been reported that the reproducibility error for breath hold techniques is about 3 mm [20]. Secondly, DTT also corrects for residual positioning errors which have been reported also to be ca 3 mm for liver cancer [21]. Thus, a residual tracking error of 1.67 mm can still be considered as an overall improvement for treatment. The errors obtained in this study are also in agreement with previously reported values [19] [18]. Depuydt et al. [19] reported a 90th percentile for the 2D tracking error in beam-eye-view as 3.08 mm which is in close comparison to the 3.33 mm determined in this study for the 3D model error.

Probable causes for the deterioration of the model quality are baseline shifts (*p* < 0.001 for correlation with number of needed models) or shifts between abdominal and thoracic breathing [13, 22].

On median 173 kV images were taken every fraction during treatment. In 64.6% of these images the defined FMs were detected. Although the detection rate is not very high, it is still sufficient to perform a DTT treatment. Fortunately, a successful automatic detection of the FM is not a requirement for the treatment, since the gimbal position is determined by the 4D model based on the superficial IR markers. Nevertheless, a high detection rate means that the Vero system is able to verify the model quality frequently and quantitatively. If the detection rate is low, the operator has to verify the model quality visually from the acquired kV images and thus ensuring the accuracy of the treatment. In this case, no quantifiable information about the model quality is available during the treatment and more focus of the operator is required. Therefore, a high detection rate is still desirable although it is not a strict requirement for the treatment.

An analysis of the additional imaging dose due to the regular kV images has been performed by Depuydt et al. [19]. In their study, images are acquired with 0.5 Hz during treatment instead of 1 Hz which is used in this study or by Makumoto et al. [23]. They found that the maximum skin dose should be below 30 mGy per treatment fraction. An analysis of the imaging dose during a DTT treatment in the University Hospital Erlangen is currently ongoing.

Since all patients have two FM implanted, both will be defined within the ExacTrac system before the first fraction. During the definition, the distance between both end points is given as a feedback. For straight markers this can prove helpful, but in the case of bent markers this distance is not well known and the end points may be difficult to see due to metal artifacts. At the beginning of the first fraction, the FM detection can be tested by taking a single X-ray image and adapted until the FM are detected within the kV image. Nevertheless, depending on the position and shape of the FM it is possible that the detection of one marker is more unreliable compared to the second one. In this case an adaptation of the marker definition can be performed, or the FM definition can be removed from the ExacTrac system entirely. If the definition of one of the two markers is removed from the ExacTrac, the DTT treatment can still be delivered, since one FM is sufficient for the ExacTrac system to track the target movement. This happened for patients 204, 206, 211, 212 and 213 and was performed by a medical physicist present during the treatment. Since the strong windowing is used during the FM definition no re-definition was needed. Of the 26 implanted markers, 6 were bent in a way that made them unusable for the marker detection. However, every patient had at least one straight marker which could be used during treatment.

For some patients it is possible that there are strong radio-opaque structures in close vicinity to the FM that can lead to problems in the marker detection from certain gantry angles (e.g. the spine in *imager1* within Fig. 1b). In these cases it can be helpful to close the jaws of the two X-ray tubes so that only a small region surrounding the markers remains visible within the kV images. The jaws are visible as dark borders within the acquired kV images (see Fig. 1b). By removing marker-like structures with the jaws the marker detection becomes more stable and reliable. Besides the advantages for the FM detection there is obviously the dosimetric advantage of closing the jaws.

In Fig. 3 one can see the distribution of the successful and unsuccessful FM detection depending on the surrounding image intensity *I* as well as the relative angle *θ* between the FM and the imager. From the resulting proportion of successful detections, it can be seen that the FM detection benefits from high intensities surrounding the marker within the kV images. An example for different intensities can be seen in Fig. 1b where the surrounding intensity within *imager1* (left) is 1735 and 5257 in *imager2* (right). This effect shows that radio-opaque structures along the line of sight with the FM lead to a decreased success rate for the detection. Considering the angular dependency, no trend can be observed for the detection probability. Nevertheless, a decrease of roughly 10% can be seen for *θ* < 10°. In this area, both FM endpoints are very close to each other, or even overlapping.

The probability of a detection in only one of the two imagers shows no visible dependency on either the relative angle or the surrounding intensity. Furthermore, no differences between the two imagers could be observed.

From the recalculation of the 4D model it can be concluded that the correlation between the external superficial movement and the internal liver movement changes significantly over the course of the treatment. The predicted tumor movement amplitudes vary approximately 4 mm around the median position for every direction. Based on this result it can be concluded that it is not possible to determine only a single correlation model (e.g. at the first treatment fraction or even from a 4DCT) for the course of the entire treatment. Furthermore, the results show that it is not possible to rely solely on external signals or surrogates to determine the internal tumor position. It is important to either monitor the tumor motion directly or to verify the used correlation model regularly. Although the mean period of validity for the correlation models in this study was 4:50 min, verifying the correlation model in an interval of at least 0.1 Hz seems recommendable since the monitoring interval also determines the maximal possible timespan of irradiation with large deviations between the true target position and the model position. To verify the target position only in the case of baseline drifts or general changes in the external signal might by insufficient, since changes in the correlation between the internal and external movement, e.g. due to changes between abdominal and thoracic breathing, would not be detected.

At the University Hospital Erlangen, the clinical experience with DTT treatments is very positive. The fact that the tumor movement can be observed during treatment is received as very positive and seen as a big improvement to the command based breath-hold treatment during which the compliance of the patient is observed with in-room cameras. Furthermore, a reduction of the clinical workflow could be noticed at our institute. Patients treated in expiration with breath-hold receive regular CTs over the course of the treatment for verifying the target position and the PTV margins. If changes are observed, the patient is being re-planned. With a DTT treatment, this step is not necessary, since the target can be observed during treatment based on the acquired kV images. Thus, several CTs and possible multiple plan changes can be spared from the clinical workflow by using a DTT treatment.

Future works include research in using IMRT as a default treatment technique and a corresponding end-to-end QA which verifies the IMRT delivery in combination with the gimbal movement. This is a current research item at our institution. Furthermore, marker-less DTT was recently introduced to the Vero with the new ExacTrac version 3.6.1. This allows lung tumor tracking without the implantation of fiducial markers which carries a large risk of pneumothorax. Unfortunately, further developments cannot be expected for the Vero system since it was discontinued by the manufacturers although there are still many possibilities which can be explored (e.g. tracking of slow moving structures without a correlation model and sparse temporal sampling of the target position which could be used for prostate cancer).

## Conclusion

Until now 13 patients with liver cancer were treated with DTT at the Vero system of the University Hospital Erlangen. The median treatment time was 19:42 min during which on median 173 kV image pairs were taken. In 64.6% of these images the marker detection was successful. An examination of the dependency of the FM detection on the relative angle towards the imager and the average surrounding intensity indicated that a high intensity in the vicinity of a FM has a positive effect on its detection whereas no effect was observed for different angles of the marker relative to the imager. Furthermore it could be shown that the number of correlation models created during treatment significantly increases in the presence of baseline shifts and that there are considerable differences between the different correlations models which were determined over the course of the treatment. Thus, the importance of regular verification of correlation models used for irradiation could be shown.

## Additional file

Additional file 1: This additional file containes the possible error codes of a failed marker detection and their respective occurence rates. (DOCX 24 kb)

## Abbreviations

3DCRT: 3D conformal radiation therapy
4D: Four-dimensional
CT: Computed tomography
DTT: Dynamic tumor tracking
FM: Fiducial marker
GTV: Gross tumor volume
HCC: Hepatocellular carcinoma
IMRT: Intensity-modulated radiation therapy
IR: Infrared
MLC: Multileaf collimator
MRI: Magnetic resonance imaging
PTV: Planning target volume
SBRT: Stereotactic body radiation therapy
