# Supplementary material for: Performance of gimbal-based dynamic tumor tracking for treating liver carcinoma
Source: Radiat Oncol. 2018 Dec 5;13:242. doi: 10.1186/s13014-018-1180-1 (PMC6280466; doi:10.1186/s13014-018-1180-1)
Supplement: Supplementary file 1 — This additional file containes the possible error codes of a failed marker detection and their respective occurence rates. (DOCX 24 kb) [file 13014_2018_1180_MOESM1_ESM.docx]

# Supplementary Materials

# *Appendix A*

# The seven most prominent error codes given within the corresponding log files are listed in table 1 together with their respective occurrence relative to the total number of kV images taken. To aid the understanding of the existing error codes a brief explanation of our understanding of the FM detection algorithm will be given:

1. The ExacTrac software searches for possible FM end points within both acquired X-ray images independently (2D candidates)
2. If several 2D candidates are found within the images, the algorithm tries to extract a 3D position of every possible end-point based on the orthogonal geometry of the kV images (3D candidates)
3. If 3D candidates are found the software compares the detected with the defined end-points based on expected distance and orientation.
4. If 3D candidates are found that match the defined marker configuration the detection was successful

*Trial detection failure interlock* : This error is not yet completely understood and therefore no countermeasures are yet known.

*not matching planned marker configuration* : If the ExacTrac is able to detected possible marker end-points within both acquired X-ray images and also 3D candidates could be calculated. However, the resulting 3D candidates do not match the defined marker configuration from the CT.

*not enough 2D candidates* : In this case it was not possible to extract enough possible end points from the given X-ray image

*MaxMarkerDistanceErrorIsom* : This error occurs if 3D candidates were found, but their distances to not match the expected distance based on the defined FM.

*not enough 3D candidates* : Although enough 2D candidates were found within the separate images, they could not be matched to a sufficient number of possible 3D candidates.

*too many 3D candidates* : Within the set of detected 3D candidates it was not possible to find an unambiguous combination of end-points that matched the defined marker configuration

*too many 2D candidates* : From the detected 2D candidates it was possible to find unambiguous 3D candidates.

**Supplementary Table 1:**

List of the error codes given of the ExacTrac and their respective occurrence.

| Error Code | Occurrence [%] |
| --- | --- |
| Trial detection failure interlock | 22.6 |
| not matching planned marker configuration | 4.4 |
| not enough 2D candidates | 3.7 |
| MaxMarkerDistanceErrorIsom | 3.2 |
| not enough 3D candidates | 1.6 |
| too many 3D candidates | 0.2 |
| too many 2D candidates | 0.1 |
